# Telehealth-delivered caregiver training for autism: Recent innovations

**DOI:** 10.3389/fpsyt.2022.916532

**Published:** 2022-12-21

**Authors:** Laura Pacione

**Affiliations:** ^1^Division of Child and Youth Mental Health, Department of Psychiatry, University of Toronto, Toronto, ON, Canada; ^2^Department of Mental Health and Substance Use, World Health Organization, Geneva, Switzerland

**Keywords:** autism (ASD), telehealth, caregiver-mediated intervention, parent training, global health, neurodevelopmental disabilities, eLearning, online training

## Abstract

Providing treatment to children with autism is a global health priority, and research demonstrates that caregivers can be trained in techniques to promote their child's social interaction, communication, play, positive behavior and skills. These caregiver-mediated interventions have been shown to promote a number of positive outcomes in children with autism, as well as their caregivers. When provided by telehealth, data indicate that caregiver training is acceptable and feasible, and associated with similar positive outcomes as live face-to-face training. Telehealth innovations, which have accelerated during the COVID-19 era, have demonstrated advantages over in-person delivery of services in terms of cost effectiveness and increased accessibility, however, more research is needed on feasibility, acceptability and effectiveness for different populations in different contexts. This brief review will highlight recent caregiver skills training interventions for autism that have been successfully adapted or designed for telehealth delivery. Telehealth interventions that are scalable, adaptable, caregiver-mediated, open-access, and delivered as part of a stepped care model, have the potential to address the global treatment gap for families of children with autism and other neurodevelopmental disabilities. Considerations relevant to the global scale-up of caregiver-mediated interventions will also be discussed.

## Introduction

Autism is a common neurodevelopmental condition affecting an estimated 1% of the population globally (1). It is characterized by difficulties in communication, social interaction and flexibility, differences in sensory processing, patterns of intense interests and repetitive behaviors (2, 3). While clinical information stresses deficits, certain strengths appear to be common in autistic individuals, including memory, attention to detail, skills related to strong special interests, and positive personality traits, including fairness and authenticity (4–7). Furthermore there is increasing recognition that valuing autism and neurodiversity benefits society as a whole (8).

Significant evidence shows that caregivers of children with autism can learn skills to promote their children's development and wellbeing (9, 10). Domains addressed by caregiver skills training include caregiver-child interaction, child joint attention, social interaction, communication, positive behavior, play, adaptive functioning, and other concerns, such as repetitive behaviors, self-regulation and sensory processing (11). Caregiver stress and lower perceived competence are common issues for caregivers of children with autism that can be improved through caregiver skills training (12–14).

Caregiver skills training is an attractive option for reducing the treatment gap globally because the majority of children with developmental disabilities, including autism, live in low- and middle -income countries where the availability of interventions is extremely limited (15). Even in high resource settings, wait times for services are lengthy, and the availability of trained personnel is a barrier (16).

The COVID-19 pandemic and the measures to contain it—including social isolation, school and workplace closures and interruptions in health and social services—have negatively affected children with autism and their families (17–19). Despite this, it is important to remember that children and families are often resilient in the face of adversity (20, 21). While causing undeniable hardship, the pandemic and the measures to contain it have also inspired innovation and propelled the use of digital technologies forward, including for training caregivers (22, 23).

This review focuses on interventions designed to train caregivers of young children with autism to support their child's development that are supported by randomized controlled trial (RCT) evidence of gains in child and/or caregiver outcomes and were adapted for telehealth delivery and published between April 2021 and Nov 2022. Selected examples of other telehealth-delivered caregiver training in Naturalistic Developmental Behavioral Interventions (NDBIs) are included to illustrate a diversity of approaches, especially those that show potential for broader scale-up.

## Caregiver-mediated interventions

Caregiver-mediated interventions are adaptations of professionally delivered interventions or interventions developed specifically for implementation by caregivers. They give children the opportunity to benefit from daily exposure to treatment techniques during regular interactions and activities with family members (24). Many caregiver-mediated interventions belong to the category of NDBIs, which are implemented through play and daily activities, informed by developmental and behavioral science, and focused on improving social communication and development (24). As a group, NDBIs share certain features, such as utilizing typical daily interactions, following the child's interest, turn taking in shared activities, imitation of the child, adult modeling of language and other skills, and natural reinforcers of the child's communication attempts and skills (24). Training caregivers to deliver NDBIs for children with autism has been proposed as a cost-effective method for reducing the treatment gap for children with developmental disabilities because it enables children to receive interventions that are otherwise unavailable due to a lack of trained professionals (24).

Programs that train caregivers to deliver NDBIs have typically been offered in person, in group or individual format and typically involve teaching skills and strategies using instruction, modeling, rehearsal, and feedback (25, 26). The use of telehealth (the provision of health services, information and education over the Internet and related technologies) offers an attractive option for scaling up care globally and has been proposed to address barriers to traditional caregiver training, including cost, lack of time, geographic isolation, lack of transportation and lack of childcare (27, 28). According to WHO, digital health technologies can improve accessibility, quality and affordability of health services, and should “complement and enhance existing health service delivery models, strengthen integrated, people-centered health services and contribute to improved population health, and health equity…” (29).

Telehealth adaptations of caregiver training programs have mainly used video conferencing software to replicate in-person delivery (30). On the other hand, some interventions, have been adapted solely for asynchronous self-directed internet-based instruction (eLearning), while others use a hybrid telehealth approach, combining telehealth-delivered caregiver coaching with self-directed eLearning and/or in-person elements. eLearning approaches that include an element of real-time remote contact with trainers has been advocated for, given that opportunities for coaching, feedback and support are associated with better outcomes (31, 32). Asynchronous telehealth interventions may include adjunctive peer-to-peer support via virtual support groups, chatrooms, discussion groups, bulletin boards or forums (33, 34) and these options should also be investigated in the context of telehealth research.

## Telehealth adaptations of evidence-based caregiver-mediated interventions

In a recent systematic review, Ellison et al. analyzed diverse telehealth interventions provided to children and adolescents with autism and their families and findings suggested that telehealth services were equivalent or better than face-to-face services (30). The review sought to extend the findings of reviews by Sutherland et al. and Boisvert et al., which reported high caregiver satisfaction with telehealth interventions (35, 36). Across studies identified by Ellison et al., the most common telehealth modality was synchronous (real-time intervention) using video conferencing software. For caregiver coaching specifically, telehealth has advantages over in-person delivery of services, including cost effectiveness and increased ability to access underserved populations (31). Telehealth-provided interventions for children with autism were also systematically reviewed by de Nocker and Toolan, revealing improvements in child level outcomes that were similar between telehealth and in-person delivery (37). Consistently, a systematic review by Pacia et al. found that caregiver-mediated interventions designed to improve social communication for children with autism that used videoconferencing and other eLearning strategies demonstrated treatment effects that were similar to in-person interventions (38). However, a recent meta-analysis of 8 RCTs of technology-assisted parent-mediated interventions for children with autism that utilized apps, websites, DVDs and computer-based interventions showed no significant differences in social communication, social functioning or language outcomes, although the majority of the interventions used technology only and did not include caregiver coaching (39).

Studies of telehealth-adapted caregiver training in evidence-based NDBIs published between April 2021 and Nov 2022 that included a caregiver coaching component and evaluated child and/or caregiver outcomes are described below. The search strategy is described elsewhere (30).

Shire et al. (40) adapted JASPER (Joint Attention, Symbolic Play, Engagement, and Regulation) for delivery using video conferencing plus 3 home visits to coach caregivers (40). The proof-of-concept study, which was conducted with caregivers of 6 children aged 2-9 years with autism who were living in rural and remote Canadian communities, found gains in children's joint engagement and caregivers' use of JASPER strategies that were similar to those seen in the face-to-face intervention, however, conclusions about effectiveness could not be made given the scope of this study. Importantly, participating families found the telehealth-delivered intervention to be acceptable and effective, and descibed the ability to connect from home and flexibility as advantages, with new technology, time, and managing other children in the home as disadvantages (40).

The Social ABCs program was adapted for telehealth delivery and tested with urban Canadian caregivers from diverse linguistic, ethnic, and educational backgrounds (41). In-person (*n* = 45) was compared to telehealth delivery (*n* = 37) for six group learning sessions and nine 60-min individual coaching sessions. Results showed good caregiver fidelity of intervention and toddler gains in social-communication skills that were similar for in-person and telehealth delivery. Importantly, the telehealth-delivered program, which included individual coaching via videoconferencing, was acceptable and feasible for a diverse group of caregivers.

Internet-based Parent-implemented Communication Strategies-Storybook (i-PiCSS) trains and coaches caregivers to use naturalistic communication teaching strategies while reading storybooks with young children with developmental disabilities (42). A single case multiple-baseline study with 3 parents used self-directed eLearning modules and coaching via videoconferencing, and demonstrated fidelity of implementation and improvements in child communication.

Preschool Autism Communication Trial (PACT) is an evidence-based communication-focused treatment for children with autism that has been adapted for use in low resource settings, and was adapted to PACT-Generalized (PACT-G), which includes caregiver- and educator-mediated components (43, 44). PACT-G was provided in a hybrid model, with both in-person and telehealth-delivered sessions. In a parallel, single-blind, an RCT, 555 children aged 2–7 years were randomized to either PACT-G (*n* = 122) or treatment as usual (*n* = 127) (45). PACT-G demonstrated improved social communication and positive effects on parental wellbeing and child disruptive behavior, although autism symptom outcomes were not different from treatment as usual and intervention dosage and poorer remote session quality were named as potential contributors to these findings (45).

WHO's Caregiver Skills Training for Families of Children with Developmental Delays or Disabilities (CST) has been tested with diverse caregivers and was developed as an open-access program for families of children aged 2–9 years to improve access to evidence-based care (46). The program, which was designed to be culturally and contextually adapted, consists of 9 group sessions and 3 home visits delivered by trained and supervised non-specialist facilitators. It has demonstrated acceptability and feasibility when delivered in-person in Ethiopia, India, Italy and Hong Kong, and virtually in Hong Kong and rural Missouri (47–51). A pilot RCT of CST in Italy (*n* = 86) demonstrated a large and significant effect on parent skills supporting joint engagement and benefits in terms of parental stress, self-efficacy, and child gestures, although changes in autism symptom severity and joint engagement were not statistically significant (52). CST was evaluated in Hong Kong and different methods of delivery were compared to waitlist control: asynchronous eLearning (digital versions of participant guides and pre-recorded videos demonstrating CST skills and strategies) (*n* = 9), videoconferencing (*n* = 7), and a hybrid model with group sessions delivered by videoconferencing and in-person home visits with coaching (*n* = 9) (49). In this study, all modes of delivery showed high acceptability and feasibility, and in-person and video conferencing were associated with greater improvements in caregivers' wellbeing and child's communication and behaviors compared to self-directed eLearning and control. An eLearning version of CST (eCST) was recently released in English on WHO's free learning platform, OpenWHO^1^, and is currently in field testing.

Caregiver training to improve child behavior has also been effectively delivered via telehealth. Collaborative Model for Promoting Competence and Success (COMPASS for Hope) delivered in 4 group and 4 individual sessions was compared for videoconference-delivery (*n* = 10), in-person delivery (*n* = 13) and waitlist control (*n* = 10), and showed similar benefits for telehealth and in-person delivery compared to control (53). Additionally, telehealth-provided Research Unit for Behavior Intervention (RUBI), a parent-mediated intervention protocol for children with autism was compared in an RCT to treatment as usual (*n* = 38, ages 21–84 months) and showed good efficacy in reducing disruptive behavior and high acceptability when provided in 12 weeks of real-time remote training and coaching (54). RUBI was also compared for face-to-face (*n* = 24) and virtual delivery (*n* = 31) in Israel with a sociodemographically diverse population and a reduction of disruptive behaviors was shown for both groups (55). Evidence also indicates that caregiver training to improve disruptive behavior is effective and acceptable when offered by telehealth in multiple countries and in low resource settings (56).

An applied behavior analysis (ABA)-based naturalistic communication intervention that was delivered by didactic training and live feedback sessions by teleconference was conducted with trainers and 5 families of children aged 2–7 years located in Ireland and Scotland. It was associated with variable gains in child communication and improved positive affect (57).

## Potential for digital innovation in the delivery of caregiver training

Recently, Gentile et al. evaluated the ATHENA telehealth program, which was delivered via an app and tablet, and included both synchronous and asynchronous sessions, modeling and parent coaching (58). The intervention, which tested caregiver outcomes only (*n* = 27) using a pre-post design, was associated with improvements in caregivers' empowerment, stress level, and behaviors to promote child learning, however, increased caregiver age was found to be a moderating factor that was associated with decreased empowerment and increased stress over the course of the intervention. The authors hypothesized that this may be due low acceptability and familiarity with technology among older caregivers, which has been proposed by others (59). This speaks to the need for a diversity of intervention approaches to meet the diverse needs of caregivers.

Wainer and Ingersoll evaluated a caregiver-mediated intervention for young children with autism, Improving Parents As Communication Teachers (ImPACT) Online, that combined self-directed internet-based instruction with therapist coaching (60). ImpACT Online consists of 12 eLearning lessons with explanatory video clips, a manual, self-assessments, interactive exercises and homework assignments. The study showed that caregivers were able to learn and use program skills and their children's spontaneous imitation skills improved. Furthermore, the hybrid model was acceptable and feasible, raising the potential that such hybrid telehealth programs that utilize self-directed and facilitator-provided elements can be used to increase access to evidence-based autism services. The same online intervention was assessed in a pilot RCT which compared self-directed eLearning vs. therapist-assisted eLearning for caregivers of children aged 2 to 6 years (*n* = 27). Both interventions showed high caregiver engagement and satisfaction but therapist assistance increased engagement and course completion (61). Child outcomes were not measured.

Ingersoll et al. randomized caregivers of children aged 1.5–6 years to receive self-directed (*n* = 13) or therapist-assisted (*n* = 14) telehealth delivery models of ImPACT Online (62). Both groups were given access to the training website and support from a technology navigator, with the therapist-assisted group receiving two 30-min telehealth sessions per week for 12 weeks. Both groups showed improved child language and caregiver self-efficacy, stress, and positive parental perceptions of their child, however, the therapist-assisted group showed improved child social skills, and greater gains in parental intervention fidelity and positive perceptions of the child (62).

Hao et al. used a quasi-experimental design to evaluate SKILLS (Skills and Knowledge of Intervention for Language Learning Success) an adaptation of Project ImPACT, in which caregivers of children aged 1–10 years chose between in-person (*n* = 15) or virtual training provided by videoconference (*n* = 15) (63). Groups were matched based on the child's age and gender, and maternal education. The intervention, which consisted of eight 1-h sessions, showed equivalent and comparable outcomes between groups, specifically, parents demonstrated fidelity of implementation and children showed gains in vocabulary and language complexity (63).

A stepped care approach to the use of telehealth interventions is increasingly being advocated for, whereby families are first offered a lower intensity intervention, and then a more intensive intervention is offered to those children and families with higher needs, certain characteristics or poorer treatment response (8). Wainer et al. conducted a small proof of concept trial in which families of children with autism ages 1.5 to 5 years (*n* = 20) were offered a self-directed eLearning program to enhance social imitation (64). Subsequently, families that demonstrated lower fidelity and no improvement in caregiver self-efficacy were directed into 5 sessions of coaching by video conference. The stepped care model showed high acceptability and feasibility with improvements in fidelity, parental self-efficacy and child social communication, although differences in child imitation ability and family quality of life were not significant.

## Considerations for global scale-up

Caregiver training that can be delivered by telehealth at scale has the potential to address global health inequities. However, the “digital divide” is used to describe the disparity between people in high-income contexts, and low- and middle-income contexts who may have limited access to internet-enabled devices, electricity, high speed internet and sufficient data to access digital interventions (65). The use of A.I.-based caregiver coaching is on the horizon and could help facilitate scale-up of caregiver-mediated interventions by mitigating the lack of skilled personnel, however, use of such advanced technologies will likely exacerbate the digital divide. There are some strategies for reducing barriers, such as making flexible digital interventions freely available through smart phone apps (65), but it will not be possible to address the treatment gap with a one-size-fits all model, and continued support of low-tech delivery of interventions, especially in certain contexts, can prevent widening of the digital divide (66). Multiple approaches will be needed to adapt existing evidence-based interventions and develop novel interventions that meet the needs of caregivers who are diverse in terms of language, culture, geography and context, including access to internet-enabled technologies (8, 62, 63).

Certain characteristics increase the scalability of evidence-based interventions and should be considered during the process of intervention design, including being manualized, deliverable by trained non-specialists, culturally and contextually adaptable, brief, affordable, group-based, and utilizing self-directed eLearning (67). Interventions need to be adaptable to the social, linguistic, economic, cultural and geographic context in which they are delivered (68–70). Some adapted evidence-based interventions may show decreased effectiveness compared to the original intervention, however, if modest, this may be acceptable given the trade-off with increased coverage and accessibility (67). At the same time, adapted evidence-based interventions should demonstrate acceptability, feasibility, relevance and cultural and contextual appropriateness across diverse populations, while also demonstrating effectiveness. The heterogeneous needs of children with autism and their families highlight the need for a stepped care approach and personalized models of intervention that consider the preferences, needs, and costs to individuals and families (8). Also, whenever possible, inclusion of children with other neurodevelopmental conditions should be prioritized in autism intervention research to potentially benefit a wider range of children and families (8).

## Discussion

Given the established body of knowledge that earlier identification and intervention leads to better outcomes (71), and the ability to detect autism in younger populations (72), the ability to scale up caregiver training and reach caregivers of newly identified children early, are critical to improving the trajectories for these children. Telehealth interventions that are scalable, open-access, caregiver-mediated and delivered as part of a stepped care approach have the potential to address the global treatment gap for families of children with autism and other neurodevelopmental disabilities. However, to address rather than exacerbate health inequities, interventions should be provided as part of universal health coverage (73). Future research should continue to assess acceptability and feasibility of telehealth interventions while acknowledging the digital divide these interventions can create (30).

Lack of data on the effectiveness of telehealth interventions remains a major barrier to implementation and scale up, therefore, larger randomized controlled studies that collect data on acceptability, feasibility and relevance, and utilize standard outcome measures when possible, are needed (30, 31, 74). Shreiberman and colleagues advocate for six research considerations to advance the development of NDBIs, including larger scale RCTs to address moderators, mediators and efficiency of treatments; measurement of functional changes in child behavior; analysis of the active ingredients within multicomponent interventions; measurements of treatment fidelity; testing treatment strategies for children who are poor responders; and use of implementation science to facilitate broad implementation (24). Furthermore, there is a need to test interventions across a range of different populations and contexts (27).

Increased investment and sustained funding for telehealth interventions is critical if these research priorities are to be realized, especially those that improve health equity, and allow interventions to reach rural, marginalized and disadvantaged families globally. Implementation of universal health coverage also requires adequate funding, political will and a commitment to the idea that all people deserve access to health (73, 75). It is also aided by the understanding that health equity benefits whole societies, not just the most marginalized (76).

Finally, it is critical to involve autistic adolescents and adults in the development and adaptation of interventions for children with autism and their families. There are now more self-identified autistic researchers and scholars in the field, although greater progress could be made in the adoption of participatory and emancipatory research methods (77). Resources for and by people with autism [see, for example, (78–80)] should be studied to determine potential benefit when included in telehealth interventions for families of children with autism. Meaningful involvement of people with autism in shaping research agendas to meet local needs has the potential to improve the relevance and impact of research and reduce health disparities for children and adults with autism and other neurodevelopmental disabilities (81–83).

## Author contributions

The author confirms being the sole contributor of this work and has approved it for publication.

## Footnotes

^1^ https://openwho.org/courses/caregiver-skills-training
